# Amazonian Plant Natural Products: Perspectives for Discovery of New Antimalarial Drug Leads

**DOI:** 10.3390/molecules18089219

**Published:** 2013-08-02

**Authors:** Adrian Martin Pohlit, Renata Braga Souza Lima, Gina Frausin, Luiz Francisco Rocha e Silva, Stefanie Costa Pinto Lopes, Carolina Borsoi Moraes, Pedro Cravo, Marcus Vinícius Guimarães Lacerda, André Machado Siqueira, Lucio H. Freitas-Junior, Fabio Trindade Maranhão Costa

**Affiliations:** 1 Instituto Nacional de Pesquisa da Amazônia (INPA), Av. André Araújo, 2936, 69067-375 Manaus, AM, Brazil; E-Mails: renatabsl@hotmail.com (R.B.S.L.); ginafrausin@gmail.com (G.F.); luizrocha_silva@hotmail.com (L.F.R.S.); 2 Departamento de Genética, Evolução e Bioagentes, Universidade Estadual de Campinas-UNICAMP, P.O. Box 6109, 13083-862 Campinas, SP, Brazil; E-Mail: stefaniecplopes@gmail.com; 3 Laboratório Nacional de Biociências (LNBio) – Centro Nacional de Pesquisa em Energia e Materiais (CNEPM) - P.O. Box 6192, 13083-970 Campinas, SP, Brazil; E-Mails: carolina.borsoi@lnbio.cnpem.br (C.B.M.); lucio.freitasjunior@lnbio.cnpem.br (L.H.F.-J.); 4 Programa de Mestrado em Sociedade, Tecnologia e Meio Ambiente. UniEVANGÉLICA-Centro Universitário de Anápolis, 75083-515 Anapólis, GO, Brazil; E-Mail: pedrovcravo@gmail.com; 5 Centro de Malária e Doenças Tropicais, LA/IHMT-Universidade Nova de Lisboa, 1349-008 Lisboa, Portugal; 6 Fundação de Medicina Tropical Dr. Heitor Vieira Dourado, 69040-000 Manaus, AM, Brazil; E-Mails: marcuslacerda.br@gmail.com (M.V.G.L.); amsiqueira@gmail.com (A.M.S.); 7 Programa de Pós-Graduação em Medicina Tropical, Universidade do Estado do Amazonas, 69040-000 Manaus, AM, Brazil

**Keywords:** herbal remedy, *Plasmodium* spp*.*, antimalarials, drug discovery, Amazonian plants

## Abstract

*Plasmodium falciparum* and *P. vivax* malaria parasites are now resistant, or showing signs of resistance, to most drugs used in therapy. Novel chemical entities that exhibit new mechanisms of antiplasmodial action are needed. New antimalarials that block transmission of *Plasmodium* spp. from humans to *Anopheles* mosquito vectors are key to malaria eradication efforts. Although *P. vivax* causes a considerable number of malaria cases, its importance has for long been neglected. Vivax malaria can cause severe manifestations and death; hence there is a need for *P. vivax*-directed research. Plants used in traditional medicine, namely *Artemisia annua* and *Cinchona* spp. are the sources of the antimalarial natural products artemisinin and quinine, respectively. Based on these compounds, semi-synthetic artemisinin-derivatives and synthetic quinoline antimalarials have been developed and are the most important drugs in the current therapeutic arsenal for combating malaria. In the Amazon region, where *P. vivax* predominates, there is a local tradition of using plant-derived preparations to treat malaria. Here, we review the current *P. falciparum* and *P. vivax* drug-sensitivity assays, focusing on challenges and perspectives of drug discovery for *P. vivax*, including tests against hypnozoites. We also present the latest findings of our group and others on the antiplasmodial and antimalarial chemical components from Amazonian plants that may be potential drug leads against malaria.

## 1. Introduction

In the absence of clinically effective vaccines, prevention and treatment of malaria critically depend on prophylaxis and drug-based therapy [1]. However, the number of available antimalarial drugs that feature distinct mechanisms of action is low. Also, the rate at which antimalarial drug-resistance is emerging and spreading is outperforming the development of new drug entities. This is especially worrying given reports of artemisinin-resistant *P. falciparum* in Southeast Asia [2,3].

In the Amazon region, plants are widely used in traditional medicine for the treatment of malaria. In 1997, Milliken surveyed the literature on plants used for the treatment of malaria infections, fevers and related conditions (headaches, *etc.*) by the people of the Amazon and Caribbean regions [4]. He described antimalarial plants that are widely distributed and used throughout the Amazon region such as *Quassia amara* L. and *Picrolemma sprucei* Hook. f. (Simaroubaceae), *Aspidosperma* spp. *Geissospermum* spp. (Apocynaceae) and *Ampelozizyphus amazonicus* Ducke (Rhamnaceae). Recently, further works have been published on the antimalarial plants and remedies used by native Amazonian groups and local populations [5,6]. Indeed, broad scope surveys of antimalarial plant components include many Amazonian plant natural products with proven antiplasmodial activity [7,8]. Many traditionally used Amazonian plants have not yet been screened for antimalarial activity and little or nothing is known about their chemical composition thus making them interesting starting points for research on new antimalarials.

In this review we highlight the potential of Amazonian plants as sources of prospective drug leads against malaria, focusing on the findings of our drug discovery group based in Manaus (Amazonas State, Brazil) and including important findings of other groups. Specifically, isolated compounds of known molecular structure from Amazonian plants for which *in vitro* or *in vivo* antiplasmodial activity has been confirmed, are covered. Despite this, several elegant studies on plant extracts, fractions and compound-enriched fractions have been published by leading research groups in the past decade [9]. Also, we discuss the currently available antimalarial drug-sensitivity assays based on *P. falciparum* for screening and drug discovery. We propose that challenges may be addressed and perspectives created in *P. vivax* drug discovery through multidisciplinary research by laboratories located outside and within the Brazilian Amazon. Specifically, in the Brazilian Amazon there is a diversity of medicinal plants that is traditionally used to treat *P. vivax* and *P. falciparum* infections. Additionally, malaria-infected patients have access to hospitals with adequate facilities and personnel where molecular, genetic and screening studies may be performed.

## 2. Malaria Drug Resistance: Urging the Discovery and Development of New Drugs

Resistance to antimalarial drugs has been described for three of the five species of human malaria parasites, *P falciparum*, *P*. *vivax* and *P*. *malariae* [10]. Resistance of *P. falciparum* to chloroquine (CQ), the most promising antimalarial of the 20th century, was first reported less than twenty years following its widespread use, with the first reports of resistance originating from South America and Southeast Asia at the end of the 1950’s [11,12]. Since then, the spread of CQ-resistance has been unrelenting, and currently, CQ-resistance is almost synonymous with *P. falciparum* [10]. In response to CQ-resistance, antifolate combination drugs were introduced. The best known and most widely used is the combination of sulfadoxine and pyrimethamine (SP) the latter being a well-known antimalarial drug often used on its own. However, pyrimethamine use had already selected resistant *P. falciparum* parasites within one year of its introduction [13]. SP combination therapy was introduced in the 1960’s but resistant parasites quickly appeared in Southeast Asia, and spread rapidly to Africa [14].

Failure of *P. falciparum* clinical treatment resulting from parasite resistance to all current antimalarial drugs has placed the new generation of drugs, called artemisinin-based combination therapies (ACTs) at the forefront of malaria control programs [15]. The rationale for ACTs is to combine a fast-acting drug (artemisinin (ART) derivative), with a partner drug with long half-life, such as mefloquine, amodiaquine, piperaquine, pyrimethamine/sulfadoxine or lumefantrine, in order to achieve rapid and effective parasite clearance and to avoid the development of resistance to ART [16]. However, resistance to the partner drug is common, and decreased efficacy to ART derivatives themselves due to resistance is already emerging [3]. The development of resistance mutations may be a rare occurrence; a spontaneous mutation may occur only once in several million parasites. Nevertheless, once it has occurred, it can be selected and disseminated. Consequently, new chemical entities that may overcome the mechanisms of resistance to currently used antimalarial drugs are in urgent demand.

## 3. Amazonian Plants: Sources of New Antimalarial Drugs Leads

A pioneer attempt to apply the scientific method to study the pharmaceutical properties of the plants from Amazonas State, Brazil was performed by the physician and naturalist Alfredo da Matta [17], whose work is still a valuable reference and starting point to research in the field. As expected in a place with such a high biodiversity, many others have been following his path, by exploring the plants used with medicinal purposes by the native communities [18,19,20]. Among the rural and riverine populations that inhabit the region, a great variety of plant families, such as Fabaceae, Arecaceae, Zingiberaceae and Lamiaceae, have been used to prevent and treat a wide variety of symptoms and diseases [19,20]. The recent discovery of plants with considerable antibacterial activity [21,22] and the repellent, larvicidal and mosquitocidal properties of plant essential oils and extracts are useful for the control of mosquito vectors of malaria and other tropical diseases and demonstrate the potential of the regional plant biodiversity [23,24] as source for new active compounds, much of which has yet to be explored. 

Plants produce useful chemicals for control and treatment of *Plasmodium* spp. that cause human malaria [25]. Historically, quinine is the most important natural product used to treat malaria. Quinine is a constituent of the traditionally used bark of *Cinchona* spp. and was isolated for the first time in the early 19th century. Synthetic quinoline antimalarials were developed during the 20th century based on knowledge of quinine´s structure [26,27]. In the 1970’s, the natural product artemisinin was isolated by Chinese scientists from the traditionally used antimalarial species *Artemisia annua* L.. Today, artemisinin is isolated from *A. annua* on an industrial scale. Semi-synthetic derivatives containing the intact 1,2,4-trioxane antimalarial chromophore moiety are prepared from artemisinin in simple reaction sequences [27]. The ACTs that have been introduced in the past two decades contain a synthetic quinoline antimalarial (a phytochemical mimic) and an artemisinin derivative (derived directly from an isolated phytochemical) [16]. As has been emphasized, traditionally used plants are the sources of the most important drugs in the current therapeutic arsenal for combating malaria. 

## 4. Field Installed Capacity and Recent Findings from the Manaus Drug Discovery Group and Others

Based on the concept that the next generation of antimalarial drugs may also originate from plants used in traditional medicine, a group of collaborators from the Tropical Medicine Foundation Dr. Heitor Vieira Dourado (FMT-HVD), the Brazilian State-owned Agronomic and Husbandry Research Company (Embrapa), the Federal University of Amazonas (UFAM) and the Federal University of Rio Grande do Norte (UFRN) under the leadership of the National Institute for Amazon Research’s (INPA’s) Laboratory of Amazonian Active Principles (LAPAAM) has focused on studies on the chemical composition and biological activity of antimalarial plants from the Amazon. Table 1 summarizes information on the chemical composition of plants from the Amazon region.

**Figure 1:**
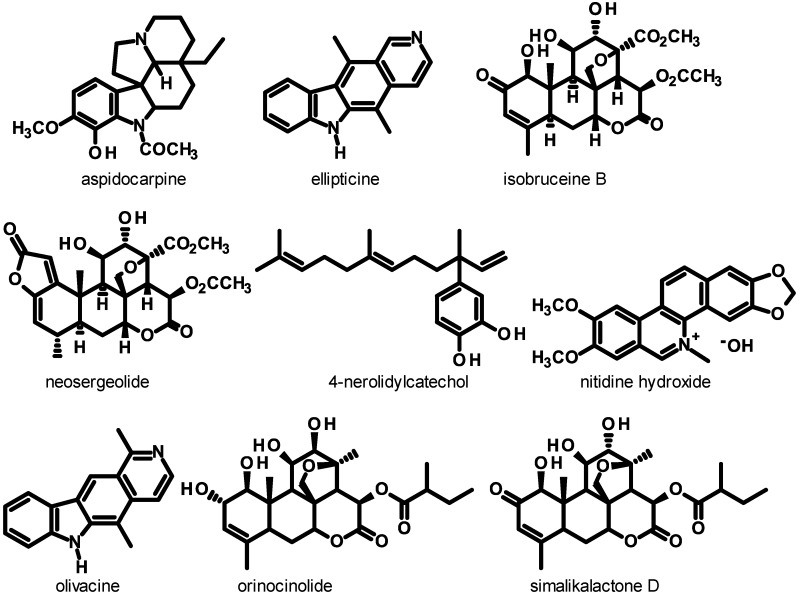
Molecular structures of antiplasmodial substances found in Amazonian plants exhibiting submicromolar IC_50_ values against *Plasmodium falciparum in vitro* and/or significant *in vivo* activity in rodent malaria models.

Several antimalarial phytochemicals may be isolated on gram or multi-gram scales thus potentiating their pharmacological study. The quassinoids neosergeolide and isobruceine B (Figure 1) have each been isolated from the stems and roots of the caferana plant (*Picrolemma sprucei*) on gram scales using a multi-stage extraction, chromatography and recrystallization sequence [28]. In other work, 4-nerolidylcatechol (4-NC, Figure 1) was readily isolated on a multi-gram scale from the roots of *Piper peltatum* L. (syn. *Pothomorphe peltata* Miq.) by straightforward extraction-chromatography. Interestingly, 4-NC makes up >5% of the dry weight of *P. peltatum* roots [29] and *P. peltatum* can be cultivated in the Brazilian Amazon where its roots produce *ca*. 27 kg of 4-NC per hectare [30,31], thus allowing the use of 4-NC in industrial applications.

**Table 1:** Amazonian antimalarial plants and their antiplasmodial chemical components.

Plant species	Family	Common name	Collection place	Part used	Active component	IC_50_ (μM)	*Pf* strain	Source
*Aspidosperma desmanthum* Benth. ex Müll. Arg.	Apocynaceae	araracanga	Brazil	bark	aspidocarpine	0.019	K1	[29,32]
*Aspidosperma ulei* Markgr.	Apocynaceae		Brazil	root bark	uleine, olivacine	1.2–3.7	K1, 3D7, W2	[33,34,35]
*Aspidosperma vargasii* A. DC.	Apocynaceae	amarelão	Brazil	bark	ellipticine	0.073–0.81	K1, 3D7	[29,33]
*Carapa guianensis* Aubl.	Meliaceae	andiroba	Peru	seed/flower oil	gedunins, an andirobin, mexicanolides, phragmalin-type limonoids (andirolides)	2.5–15	FCR-3	[36]
*Moronobea coccinea* Aubl.	Clusiaceae	manniballi	French Guiana	latex	polyprenylated acylphloroglucinols, isogarcinol,cycloxanthochymol, garcinol	2.1–37	FcB1	[37]
*Picrolemma sprucei* Hook. f.	Simaroubaceae	caferana	Brazil	root, stem	isobrucein B, neosergeolide	0.002–0.008	K1	[29,38]
*Piper peltatum L.*	Piperaceae	caapeba	Brazil	root, leaf, flowering part	4-nerolidylcatechol	0.67	K1	[29]
*Quassia amara* L.	Simaroubaceae	quinquina de Cayenne	French Guiana	fresh leaf	simalikalactone D, simalikalactone E	0.010–0.068	FcB1, F32, W2	[39,40]
*Rheedia acuminata* (Ruiz & Pavon) Planchon & Triana	Clusiaceae	cerillo, botoncillo, caraño	French Guiana	root bark	prenylated xanthones, polyprenylated acylphloroglucinols	3.2–15	FcB1	[37]
*Simaba orinocensis* Kunth	Simaroubaceae		Peru	root bark	orinocinolide, simalikalactone D	0.0063–0.018	D6, W2	[41]
*Tabebuia incana* A.H. Gentry	Bignoniaceae	pau d´arco	Brazil	bark	5 & 8-hydroxy hydroxyethyl naphtho[2,3-b]furan-4,9-diones	0.67	FcB2	[42,43]
*Tachia grandiflora* Maguire & Weaver	Gentianaceae	caferana	Brazil	leaf	amplexine (djalonenol)	35	W2	[44,45]
*Tapirira guianensis* Aubl.	Anacardiaceae	piojo	French Guiana	bark	cyclic alkyl polyols	4.7–5.4	F32, FcB1	[46]
*Zanthoxylum. rhoifolium* Lam	Rutaceae	tachuelo	French Guiana	trunk bark	avicine hydroxide, nitidine hydroxide, fagaridine	<0.27–38	FCB1	[47]

Hyphenated and other analytical methods have been used to determine isobrucein B and neosergeolide in caferana (*Picrolemma sprucei*) infusions [28,48], 5 and 8-hydroxy-2-(1-hydroxy- ethyl)naphtho[2,3-b]furan-4,9-dione concentrations in pau d´arco (*Tabebuia incana* A.H. Gentry) bark infusions[42] and 4-NC in *Piper peltatum* root infusions [30]. UPLC-ESI-MS analysis of the bark infusions of 8 *Aspidosperma* spp. from Manaus, Brazil revealed that only *A. vargasii* contained ellipticine and 2-methyl-1,2,3,4-tetrahydroellipticine [49].

LAPAAM in collaboration with others has been actively screening plant extracts for antimalarial activity. Since 2007, *in vitro* testing for antimalarial activity by LAPAAM/FMT-HVD has been based on direct counting the number *P. falciparum* in at least 1,000 erythrocytes on thick blood smears using optical microscopy. Several methods (ELISA and tritiated hypoxanthine) are being adopted by INPA to increase screening capacity of plant extracts in the future. Several infusions prepared from carapanaúba tree (*Aspidosperma* spp.) bark collected in the region around Manaus exhibited strong *in vitro* inhibition against *Plasmodium falciparum* K1 strain [32]. In other work, MeOH extracts of acariquara branca (*Geissospermum argenteum*) from Roraima State were also active (IC_50_ = 4.6 μg/mL) against the K1 strain of *P. falciparum in vitro*. However, acariquara vermelha (*Minquartia guianensis*) extracts were inactive (IC_50_ > 50 μg/mL) [50]. CHCl_3_ fractions obtained from root MeOH and leaf EtOH extracts of the caferana plant (*Tachia grandiflora* Maguire & Weaver) inhibited *P. falciparum* (IC_50_ = 11 and 36 μg/mL, respectively). Root infusions (500 mg/kg/day) of *T. grandiflora* administered orally inhibited (45%–59% inhibition) *Plasmodium berghei* in infected mice [44]. EtOH and H_2_O root extracts and piceatannol and chrysophanol isolated from roots of the mari-mari-da-terra-firme (*Cassia spruceana* Benth.) [31] were inactive against the K1 strain of *P. falciparum* [51]. Infusions of *Artemisia annua* cultivated in different Amazonian ecosystems and in another region of Brazil strongly inhibited the K1 and 3D7 standard strains and Amazonian field isolates of *P. falciparum* [33].

Several of the isolated phytochemicals in Table 1 and derivatives of these natural compounds exhibit significant *in vitro* and *in vivo* antimalarial activity. In a seminal publication by our group, the indole alkaloids aspidocarpine (Figure 1, IC_50_ = 19 nM) and ellipticine (IC_50_ = 73 nM), the quassinoid neosergeolide (IC_50_ = 2.0 nM) and 4-NC (IC_50_ = 0.67 μM) significantly inhibited the K1 strain of *P. falciparum* [29]. Neosergeolide (Table 1) is toxic to human leukemia cells *in vitro* but is not toxic to normal cells [52]. The cytotoxicity parameters of neosergeolide and isobrucein B against normal human cells requires further evaluation. Semi-synthetic derivatives 12-acetylneosergeolide (IC_50_ = 0.22 μM) and 1,12-diacetylisobrucein B (IC_50_ = 37 μM) were much less active *in vitro* against the K1 strain of *P. falciparum* than neosergeolide (IC_50_ = 7.9 nM) and isobrucein B (IC_50_ = 2.1 nM) from which these derivatives were prepared [28]. 

Other important natural products from Amazonian plant sources are also presented in Table 1. One of the most significant is the quassinoid simalikalactone D which has been isolated from *Simaba guianensis*, *S. orinocensis* and *Quassia amara* among other species of Simaroubaceae. Its antiplasmodial activity was first described in 1993 and it was repeatedly shown to present high antiplasmodial activity against *Plasmodium falciparum* strains [53]. Simalikalactone D was also found to have a median effective dose (ED_50_) of 3.7 mg/kg/day against the rodent malaria parasite *Plasmodium yoelii yoelii*, highlighting its therapeutic potential *in vivo* [39]. 

In other work, limonoids from *Carapa* *guianensis* have been shown to have significant *in vitro* antiplasmodial activity against *P. falciparum* (Table 1), with no selective toxicity against mouse mammary tumor cells [36]. The cytotoxicity of these limonoids should be studied further against normal human cell lines. Furthermore, given the *in vitro* and *in vivo* antimalarial potential demonstrated by the limonoid gedunin and its semi-synthetic derivatives, it is important to study the activity of *C. guianensis* gedunin structurally-related isolates in *in vivo* rodent malaria models.

The significant *in vitro* antiplasmodial activity of ellipticine (Figure 1) against *P. falciparum* has been independently confirmed [45]. Also, some simple derivatives of ellipticine exhibit greater *in vitro* activity than ellipticine against *P. falciparum* [54]. At LAPAAM, derivatives of ellipticine have been prepared by direct reactions on ellipticine, however none of these simple ellipticine derivatives exhibited greater *in vitro* inhibition against *P. falciparum* than ellipticine itself [49]. In a comparative study, we found that ellipticine (IC_50_ = 0.35–0.81 μg/mL) exhibited greater *in vitro* activity against the K1 and 3D7 strains of *P. falciparum* in general than its natural structural isomer, the indole alkaloid olivacine (Figure 1IC_50_ = 1.2–1.4 μg/mL) and greater *in vitro* activity than the indole alkaloid cryptolepine (IC_50_ = 0.80–0.91 μg/mL) [33]. Cryptolepine exhibited elevated *in vitro* toxicity to murine macrophages, whereas ellipticine and olivacine exhibited low toxicity to macrophages and selectivity indices >2800. Also, ellipticine (10 mg/kg/day) administered orally or subcutaneously for 4 days was the most active compound tested. It significantly inhibited *P. berghei* in mice (inhibition of 70%–77% *versus* non-treated controls) and led to mean survival times (MST) of 27–29 days for infected mice. At 50 mg/kg/day, ellipticine exhibited comparable *in vivo* activity to that of the positive control substance chloroquine. Olivacine administered orally was also a highly effective inhibitor of *P. berghei* in mice at ≥50 mg/kg/day providing long MSTs [33]. Ellipticine, olivacine and similar highly planar antiplasmodial compounds may inhibit *P. falciparum* growth through a parasite-specific mechanism-involving blockage of the formation of hemozoin (malaria pigment) through formation of stable complexes with heme in the parasite digestive vacuole [55]. We believe that ellipticine and related structural isomer olivacine may be important prototypes for the development of a new class of indole alkaloid antimalarials. More derivatives and analogs of ellipticine should be screened for *in vitro* and *in vivo* antiplasmodial activity to establish SAR parameters and optimal structures.

We further investigated the *in vitro* and *in vivo* antimalarial activity of 4-NC and also that of semi-synthetic derivatives of 4-NC. We characterized their activity against the K1 (IC_50_ = 1.9 μM) and 3D7 (IC_50_ = 6.7 μM) strains (chloroquine resistant and non resistant strain, respectively) and two Amazonian field strains (IC_50_ = 0.16–2.6 μM) of *P. falciparum in vitro* and against *P. berghei* in mice. *In vivo* suppression of *P. berghei* in the 4-day suppressive test was observed by oral administration (60%–63% on days 5 and 7 after infection) and by subcutaneous administration (61% on day 7 after infection) at doses of 600 mg/kg/day. Also, despite low activity against *P. berghei in vivo*, after ingesting 4-NC, the blood serum of healthy mice was quite active *in vitro* against *P. falciparum* [56]. *O*-mono and *O,O*-disubstituted semi-synthetic derivatives of 4-NC were prepared and found to inhibit (IC_50_ = 0.67–23 μM) the K1 strain of *P. falciparum in vitro* [38]. These derivatives are more stable than 4-NC and exhibit significant antioxidant activity [57]. Importantly, the *O*,*O*-diacetyl derivative of 4-NC at oral doses of 10–25 mg/kg/day in the 4-day suppressive test inhibited *P. berghei* in mice by 67%–70% on days 5 and 7, making this derivative *ca*. 60 times more active than 4-NC (Rocha e Silva, Pohlit *et al.* unpublished data). Many terpenes from plants exhibit activity against *Plasmodium* spp. [25,58] and it is believed that the inhibition by 4-NC and its derivatives is due in part to the 15 carbon terpene nerolidyl side-chain common to these compounds (and the antioxidant properties of 4-NC and derivatives). There is experimental evidence that *O*,*O*-diacetyl 4-NC, like many terpenes found in plants, inhibits the synthesis of terpenoid-containing metabolites in *Plasmodium falciparum* (Rocha e Silva, Katzin, Pohlit *et al.* unpublished data). In the future, more derivatives of 4-NC should be synthesized on gram-scales for evaluation of their *in vivo* antimalarial properties in rodent models, as well as toxicological evaluation necessary for further development.

In conclusion, we envisage that the plant-derived compounds highlighted above hold great promise as effective antimalarials. However, from a drug development perspective there are several gaps that need to be addressed before these compounds are considered for clinical development. Further studies on their pharmacokinetic properties, namely on absorption, distribution, metabolism, excretion and toxicity (ADMET) are desirable in order to best understand the steps required for chemical modifications towards the synthesis of analogues with improved properties. The synthesis of analogues with enhanced efficacy would also benefit from in-depth studies on the mechanisms of action of these compounds. This information would provide important biochemical clues about drug-target affinities, which would pave the way for the chemical modifications required for improved potency. The route of drug administration *in vivo* should be considered more often, as there may be variable pharmacokinetic outcomes that will impact on the drug’s efficacy depending on the drug’s entry route. Finally, if the above parameters are carefully articulated it may be possible and highly desirable, that the *in vivo* potency of these compounds in experimental models may improve by 10 to 100-fold, making them serious candidates for the next steps of drug development.

## 5. New Generation Antimalarials: Desirable and Essential Characteristics

New antimalarial drug leads should be active against all known malaria parasites that infect humans (*P. falciparum*, *P. vivax*, *P. malariae*, *P. ovale* and *P. knowlesi*) [1,59]. Differences in the biology and life cycles of these species can make this a challenging requirement. New drugs should selectively affect *Plasmodium* spp. while exhibiting tolerable toxicity profiles in humans. Preferably, these compounds should target *Plasmodium* spp. via parasite-specific modes of action involving molecular targets and organelles found exclusively in these protozoans [60]. 

Many common antimalarials were developed to be effective against blood stages of *Plasmodium* spp. New antimalarial drug leads should ideally be active against blood stages of diverse *Plasmodium* spp. and block transmission of gametocytes of *Plasmodium* spp. from humans to female *Anopheles* spp. mosquitos (malaria vectors) during blood meals (transmission-blocking activity). Also, new chemical entities are desirable that inhibit *Plasmodium* spp. development prior to or in the liver stage (prophylaxis) [1].

## 6. Drug Discovery for *P. falciparum*: The Path to Appropriate Drug-Sensitive Assays

The first *in vitro* test to assess *P. falciparum* drug-sensitivity was developed in 1968 by Rieckmann *et al.* [61] and consisted in evaluating parasite development from early ring stage to mature schizonts. After 24 h of incubation with or without drugs (controls), schizonts were counted in relation to the total number of erythrocytes on thick blood smears prepared from the cultured samples [61]. This technique known as ‘macrotest’ was capable of distinguishing between chloroquine-sensitive and resistant parasite isolates [61]. The schizont maturation test was improved after the establishment of the continuous culture in 1976 and the ‘microtest’ developed still remains one of the most common techniques for the assessment of drug sensitivity *in vitro* [62]. 

This schizont maturation assay is relatively simple to perform. It requires no expensive equipment. It is, however, a labor-intensive procedure and it needs highly trained microscopists [63]. Importantly, this test might also result in misinterpretation of data, as drugs that delay parasite growth might give the same results as drugs that completely inhibit the parasite development [63].

Soon after the introduction of continuous *in vitro* cultivation of *P. falciparum* over 30 years ago [64], several methodologies were developed to assess *P. falciparum* growth *in vitro*. Later, standardizing of antimalarial drug screening was achieved. Typically, synchronized parasites at ring stage are cultured in the presence of different concentrations of test compound or medium (control). After one schizogonic cycle (48 h), parasite growth is determined by different methods and IC_50_ values can be assessed by linear regression analyses of the linear segments of the dose-response curves. Alternatively, unsynchronized cultures or more hours of incubation can be employed [63].

The oldest and also the most low cost method for assessment of *Plasmodium falciparum* growth *in vitro* is counting giemsa-stained parasites by light microscopy (the World Health Organization standard method). Although simple, this technique is time-consuming and relies on well-trained microscopists. It allows for the testing of only a small number of compounds in a given period of time. To overcome those limitations, tritiated hypoxanthine uptake by parasites has been widely employed [65]. Hypoxanthine is taken up by the parasite as part of purine salvage and DNA synthesis that are essential for parasite development and multiplication. Although highly reliable, this standard method is expensive and uses radioactive materials with limited availability, safety, and with a strict disposal regulation in mostly developing countries [63,66].

*P. falciparum* development can also be determined by means of the enzymatic activity of pLDH (parasite lactate dehydrogenase). pLDH is a terminal enzyme in the glycolysis pathway of the malaria parasite. In the pLDH assay, nitroblue tetrazolium is reduced forming a blue formazan product that can be measured by spectrophotometry [67]. This assay requires initial parasite densities of 1%–2% and therefore, its use is limited [63]. A new pLDH-based assay that measures pLDH levels in a double-site enzyme-linked LDH immuno-detection (DELI) assay has been developed. This method is considered to be more sensitive [68] than previous methods and therefore is applicable in field conditions. Another ELISA-based test relies on the measurement of a histidine and alanine-rich protein produced by *P. falciparum* in the course of its growth and multiplication (HRP2 assay) [69]. The HRP2 assay uses a longer culture time than most other assays (72 h instead of 48 h) and parasite growth is assessed by measuring the production of HRP2 in a simple, commercially available, double-site sandwich, ELISA test kit or essentially any ELISA that is specific to HRP2.

Many nucleic acid dyes (SYBR Green I, YOYO-1, propidium iodide, acridine orange, hydroethidine, Hoechst, DAPI) have been used to assess antimalarial activity of a variety of compounds. These methods take advantage of the fact that human erythrocytes lack DNA and RNA [66]. Many methods are based on the use of the nucleic acid dyes and, have now been used in high throughout put screening (HTS). These methods vary from a simple fluorescence-based parasite proliferation assay [70] to a nuclear dye-based high- throughput confocal imaging assay [71]. Flow cytometry is another method that uses nucleic acid dyes and has the advantage that appropriate gating can also allow one to distinguish different parasite erythrocytic stages [72,73]. Transfected parasites expressing nucleic acid dyes appear to be a useful model for drug screening despite the greater complexity of this method. Green fluorescent protein (GFP)-recombinant *Plasmodium* parasites have been successfully used in antimalarial screening [74]. Aside from assays using nucleic acid dyes, transfected parasites expressing firefly luciferase are another approach for assaying antimalarial drugs. This method generates similar sensitivity results to radiolabeled hypoxanthine uptake [75,76].

Lastly, to accelerate drug discovery we have recently established a high-throughput high-content assay to monitor the intraerythrocytic asexual cycle of *P. falciparum*. The assay consists of imaging a synchronized population of parasites during the erythrocytic cycle and determining through the size of fluorescently labeled organelles the developmental age of the parasite. An automated algorithm analyzes the images, differentiating between rings, trophozoites and schizonts. This method allows for high throughput screening in an unbiased way. It permits the determination of the stage(s) in the erythrocytic cycle in which the drug is active. It is now being used to investigate the mechanism of action of novel antimalarials (Freitas Junior and Ayong, unpublished data). 

It is important to note that the *in vitro* techniques based on *P. falciparum* cultures do not provide information on potential *in vivo* metabolism of phytoconstituents that may be required as an activation step of prodrug antimalarial substances. Also, pharmacodynamic, pharmacokinetic and toxicologic data are not forthcoming from *in vitro* studies. Phytoconstituents exhibiting promising *in vitro* antimalarial activity must therefore be evaluated for *in vivo* antimalarial activity against *P. berghei*, *P. chabaudi*, *P. yoelii* or other rodent or animal models of malaria [77]. *In vivo* studies are sometimes difficult to perform given that larger quantities of isolated natural products are frequently not available. Also, government restrictions adopted in many countries to avoid a deliberate use of a large number of animals are also a factor to be taken in consideration. However, *in vivo* studies are essential for the development of antimalarial drugs from phytochemicals.

## 7. *Plasmodium vivax* Malaria: Neglected and Misinterpreted

*Plasmodium vivax* threatens 2.48 billion people worldwide and infects between 130 and 435 million people per year [78,79]. *P. vivax* has some characteristics that make it harder to control than *P. falciparum*, including the existence of dormant liver forms (hypnozoites) that cause relapses and the relatively early presence of circulating gametocytes [80]. Accordingly, *P. vivax* is becoming proportionately a more common cause of malaria in some areas where *falciparum* malaria control has been strengthened [81,82,83]. In Brazil, for example, *vivax* malaria became predominant around 1990, when control measures were intensified [84]. In 2011, Brazil has reported more than 247 thousand malaria cases (due to *P. vivax*, *P. falciparum* and *P. malariae*), with 87% of them attributed to *P. vivax* [85].

The dogma that classified *P. vivax* infection as a benign disease is now largely discredited. This is due to increasing reports of severe disease caused by this parasite combined with reassessment of the importance of the morbidity and mortality associated with this parasite to public health. Indeed, there are a great number of studies focusing on severe manifestations of *P. vivax* worldwide [86,87,88,89,90,91,92,93,94,95,96,97,98,99,100]. This former misconception has contributed to long-term neglect of *P. vivax* infections. One of the consequences of this neglect has been that little effort towards research and development of new anti-*P*. *vivax* drugs has been made [101,102,103].

Treatment of *P.* *vivax* infections in most endemic areas is based on chloroquine (CQ), used alone or in combination with primaquine. The latter is the only approved antimalarial drug that acts on *Plasmodium* spp. liver stages (hypnozoites), preventing relapses [85]. *P. vivax* CQ-resistance was reported in Papua New Guinea in 1989 [104], almost thirty years after the emergence of CQ-resistance in *P. falciparum* [105]. To date, there is clear evidence of the existence of CQ-resistant *P. vivax* in many other countries, including those of northern South America [106,107,108]. The high prevalence of CQ-resistant *P. vivax* in some areas [106,109] has been hypothesized as an important contributor to the high risk of severe *vivax* disease [88] and contributes to the overall morbidity.

## 8. Drug Discovery for *P. vivax*: The Next Step Forward

*P. vivax* infection is a neglected disease for which few drug susceptibility studies have been performed [103]. Indeed, drug susceptibility assays are a complicated undertaking as long term *in vitro* culture systems for the erythrocytic forms of this parasite have not yet been developed. This technological barrier precludes the implementation of *P. vivax* drug discovery programs. Efforts towards discovery, characterization and proper validation of *P. vivax* drug targets are significantly hampered. Knowledge of these molecular targets is necessary for the development of specific biochemical assays. Thus, medium to large scale screening campaigns of compound libraries against whole *P. vivax* parasites are not possible. Such campaigns would require robust and reproducible assays using lab-adapted *P. vivax* parasites. From the clinical drug development perspective, major candidate molecules against *P. falciparum* have been proposed to be tested first in proof-of-concept studies using *P. vivax*-infected patients, since this infection is considered to be more benign and therefore, phase II studies would not submit patients to higher risk of severe disease. However, the increase in the report of severe *vivax* cases worldwide makes this approach somehow questionable. 

In this scenario, the pragmatic solution is to continue to use *P. falciparum* as a surrogate model for *vivax* antimalarial drug discovery (see section on drug-sensitivity assays). Historically, clinical use of the same drugs to treat all human malarias has provided experience that in spite of biological differences, *P. falciparum* is a suitable model for *in vitro* drug testing and drug discovery for *P. vivax* malaria. However, reports in the past years have challenged this belief. For example, it has been suggested that *P. vivax* is intrinsically resistant to antifolates [110], although this has been contested by more recent data [111]. Russell *et al.* demonstrated that *P. vivax* trophozoites are considerably more resistant to chloroquine than *P. falciparum* trophozoites in *ex vivo* maturation assays, even when parasites were sampled from patients from endemic areas associated with reasonable chloroquine therapeutic sensitivity [112]. Additionally, experimental data suggest that mechanisms of resistance to chloroquine differ between *P. falciparum* and *P. vivax* [111,113,114].

The *ex vivo* schizont maturation test has been successfully employed over the past years to assess levels of antimalarial resistance and test novel drug candidates against *P. vivax* [115,116,117,118]. This assay consists of visually monitoring the erythrocytic development of ring and young trophozoites into mature schizonts, and drugs that are able to inhibit the maturation process in a dose-dependent manner are considered active against *P. vivax*. However, the test results can be influenced by the initial blood stage composition and the speed of maturation of the clinical isolate [112,116]. In any case, though it is not a high throughput method, the 48 h short-term *in vitro* culture of *P. vivax* may serve at the moment as a reasonable screening tool in order to evaluate antimalarials against this species’ blood stages. 

## 9. Hypnozoite Drug Tests: The Greatest Challenge

To achieve malaria eradication it is paramount to have drugs effective against hypnozoites, the mysterious dormant liver stage that causes relapses in *P. vivax* and *P. ovale* malaria [119]. To date, few *in vitro* hypnozoite assays have been developed for both *P. vivax* and the monkey malaria parasite *P. cynomolgi* [120,121,122,123]. However it remains to be shown whether these assays can be used for routine drug discovery as they are technically challenging, and can only be carried out in facilities where insectaries are available. Also, in the case of hypnozoites, as opposed to blood stages, the use of *P. falciparum* as surrogate model is unsuitable, as it cannot produce hypnozoites. In the Amazon, in some research centers, the possibility of inducing experimental *P. vivax in vitro* liver stage infection (concomitant availability of infected patient-derived gametocytes and insectaries with *An. aquasalis* in Manaus, Brazil or *An. albimanus* in Cali, Colombia) paves the way for future studies, which are not possible outside the endemic area.

## 10. Perspectives

In the future, we believe that several substances derived from Amazonian plants could be evaluated for *in vitro* activity against clinical samples of *P. vivax* in limited culture and for transmission-blocking and prophylactic activity of *P. berghei* in different laboratory models. As we mentioned, ellipticine, its derivatives and 4-nerolidylcatechol derivatives have potential as lead compounds for the development of new antimalarial drugs.

*P. vivax* has tended to be neglected despite recent reports indicating that severe *vivax* malaria may be underestimated. Moreover, there is evidence that *P. vivax* has developed resistance to chloroquine, sulfadoxine + pyrimethamine [124] and primaquine [125]. There is now a priority to also put emphasis on *P. vivax*-directed research. Thus, the use of novel screening technologies, most notably image-based assays that can distinguish between the live and dead parasites and between rings, trophozoites and schizont stages (Freitas-Junior and collaborators, unpublished data), and cytometry-based high-content screening with probes that can identify and quantitate mature blood stages in low parasitemia [126,127,128] hold promise if they can be coupled to the schizont maturation test. Recent work featuring an automated high content screening assay that measured parasite size and consequently schizont growth and maturation of *P. yoelii* liver stage [129] demonstrated that this approach is viable. A rational approach, given the variable nature of the *ex vivo* maturation test, would be to have antimalarial drugs tested against lab adapted *P. falciparum* strains to select only compounds able to block the intraerythrocytic development and maturation of asexual forms. In this way, the likelihood of finding compounds active against clinical isolates in *ex vivo* maturation tests would increase. Although more challenging, similar strategies could be employed to selected drug leads in *P. vivax*-infected hepatocytes. 

## 11. Concluding Remarks

Traditionally-used Amazonian plants are important for the discovery of new antimalarial leads against *P. falciparum* and *P. vivax*. The success of this drug discovery effort depends on the availability of increased capacity screening facilities in malaria endemic areas, which in turn are very often resource-limited areas. Therefore, solid drug discovery programs for *P. vivax* depend on the establishment of multidisciplinary research networks involving several institutions and *expertises*. Also, considerable investments are needed to equip laboratories in endemic regions to develop novel assays that can enable the screening of novel compounds against the *P. vivax* parasite.
